# COVID-19 threatens to cause collateral delay in cancer diagnosis

**DOI:** 10.1590/1516-3180.2020.033730062020

**Published:** 2020-08-03

**Authors:** Diego Lopes Paim Miranda, Angélica Nogueira-Rodrigues, Thales Pardini Fagundes, Ronniel Morais Albuquerque, Luciana Castro Garcia Landeiro

**Affiliations:** I Undergraduate Student, School of Medicine of Bahia, Universidade Federal da Bahia (UFBA), Salvador (BA), Brazil.; II PhD. Physician, Adjunct Professor and Researcher, Universidade Federal de Minas Gerais, Belo Horizonte (MG), Brazil.; III MD. Physician, Universidade Federal de Minas Gerais, Belo Horizonte (MG), Brazil.; IV Undergraduate Student, Universidade Federal de Minas Gerais, Belo Horizonte (MG), Brazil.; V

Dear editor,

In December 2019, a novel coronavirus disease termed COVID-19 emerged in Wuhan, China. It is caused by severe acute respiratory syndrome coronavirus 2 (SARS-CoV-2). Because COVID-19 is able to spread through respiratory droplets, the number of cases has rapidly increased in many countries throughout the world, including Brazil.^1^ The World Health Organization (WHO) has declared COVID-19 to be the first worldwide pandemic involving a coronavirus disease, which classifies this outbreak as an international emergency.^1^ In order to reducing the peak incidence of infections and hospitalizations and thus avoid overloading of healthcare systems, restrictive measures have been adopted all over the world. In an effort to conserve resources and reduce the risk of transmission, non-urgent laboratory and imaging tests, along with scheduled procedures, have been suspended across the world to help healthcare facilities in dealing with the ­COVID-19 outbreak. Thus, elective procedures may be delayed for an indeterminate period of time.^2^

In May 2020, the American Society of Clinical Oncology (ASCO) published a special report recommending postponement of any visits to clinics and any cancer screening or diagnosis and staging-related procedures if this postponement does not pose a risk for disease progression or worsening of the prognosis.^3^ It is reasonable to limit procedures and imaging or laboratory investigation for patients whose disease is only suspected clinically to present low risk of rapid progression or low risk of recurrence.^3^ On the other hand, delaying all screening, diagnostic and staging procedures will probably lead to an unprecedented elevation of cancer diagnoses at late stages in subsequent months.

There is a need to propose new screening strategies to avoid delay in recognition of cancer caused by postponement of diagnostic investigations due to prolonged COVID-19 containment measures. At this unusual phase of the pandemic, there needs to be careful evaluation of the risks and benefits of pursuing each procedure. One suggestion would be to incorporate alternatives such as self-collection of specimens for the fecal occult blood test (FOBT) or human papillomavirus (HPV) test.^4^ Health diagnostic centers could also have a specific day and/or location for performing preventive procedures, such as mammography and colonoscopy. Requests for imaging and laboratory tests could be sent directly to the diagnostic center, thus avoiding the need for patient consultation.

Through adopting such measures, in addition to those recommended by the World Health Organization (WHO) for the general population, screening and diagnostic activities can be made safer. Healthcare providers should also be alert to, and be trained to investigate, the presence of any symptom of COVID-19 infection in all patients who are referred for screening or diagnosis procedures.^5^

The return to normalcy may be slow. Strategies to ensure that cancer screening and prevention measures can be implemented need to be discussed within the major international medical societies. Through such strategies, a significant increase in late-stage disease at diagnosis and, consequently, higher cancer morbidity and mortality rates in the near future may be avoided.
